# Induction of necrotic cell death by oxidative stress in retinal pigment epithelial cells

**DOI:** 10.1038/cddis.2013.478

**Published:** 2013-12-12

**Authors:** J Hanus, H Zhang, Z Wang, Q Liu, Q Zhou, S Wang

**Affiliations:** 1 Department of Cell and Molecular Biology, Tulane University, New Orleans, LA, USA; 2 Department of Ophthalmology, University of Texas Southwestern Medical Center, Dallas, Texas, USA; 3 Department of Molecular Biology, University of Texas Southwestern Medical Center, Dallas, Texas, USA; 4 Department of Biochemistry, University of Texas Southwestern Medical Center, Dallas, Texas, USA; 5 Department of Ophthalmology, Tulane University, New Orleans, LA, USA

**Keywords:** cell death, RIPK3, RPE cell, oxidative stress, AMD

## Abstract

Age-related macular degeneration (AMD) is a degenerative disease of the retina and the leading cause of blindness in the elderly. Retinal pigment epithelial (RPE) cell death and the resultant photoreceptor apoptosis are characteristic of late-stage dry AMD, especially geographic atrophy (GA). Although oxidative stress and inflammation have been associated with GA, the nature and underlying mechanism for RPE cell death remains controversial, which hinders the development of targeted therapy for dry AMD. The purpose of this study is to systematically dissect the mechanism of RPE cell death induced by oxidative stress. Our results show that characteristic features of apoptosis, including DNA fragmentation, caspase 3 activation, chromatin condensation and apoptotic body formation, were not observed during RPE cell death induced by either hydrogen peroxide or *tert*-Butyl hydroperoxide. Instead, this kind of cell death can be prevented by RIP kinase inhibitors necrostatins but not caspase inhibitor z-VAD, suggesting necrotic feature of RPE cell death. Moreover, ATP depletion, receptor interacting protein kinase 3 (RIPK3) aggregation, nuclear and plasma membrane leakage and breakdown, which are the cardinal features of necrosis, were observed in RPE cells upon oxidative stress. Silencing of RIPK3, a key protein in necrosis, largely prevented oxidative stress-induced RPE death. The necrotic nature of RPE death is consistent with the release of nuclear protein high mobility group protein B1 into the cytoplasm and cell medium, which induces the expression of inflammatory gene *TNFα* in healthy RPE and THP-1 cells. Interestingly, features of pyroptosis or autophagy were not observed in oxidative stress-treated RPE cells. Our results unequivocally show that necrosis, but not apoptosis, is a major type of cell death in RPE cells in response to oxidative stress. This suggests that preventing oxidative stress-induced necrotic RPE death may be a viable approach for late-stage dry AMD.

Age-related macular degeneration (AMD) is the leading cause of blindness in the elderly.^1^ The late stage of dry AMD, known as geographic atrophy (GA), is characterized by extensive loss of retinal pigment epithelium (RPE) cells and the neighboring photoreceptors and choroicapillaris. Dry AMD accounts for 90% of AMD cases, whereas GA accounts for 35% of all cases of late-stage AMD and 20% of legal blindness attributable to AMD.^2^ Although the etiology of dry AMD remains unclear, oxidative stress and chronic inflammation are believed to be triggered by genetic and environmental risk factors to drive dry AMD pathogenesis. Consistent with a critical role for oxidative stress in AMD, clinical studies have shown that AMD disease progression can be slowed with antioxidant vitamins and zinc supplements.^3, 4^

RPE of the retina is essential for retinal homeostasis by transporting nutrients and waste for the retinal photoreceptor cells. The retina is particularly vulnerable to oxidative damage, which increases its reactive oxygen species production.^5, 6^ Reactive oxygen species overload can cause RPE cell death and chronic inflammation, leading to pathological immune response in AMD. The RPE death mechanism in AMD by oxidative stress is still controversial, with most pointing to a dominant role for apoptosis.^7, 8^ Most *in vitro* data also attribute apoptosis as a major mechanism of RPE cell death in response to prooxidants, including hydrogen peroxide (H_2_O_2_) and its stable form *tert*-butyl hydroperoxide (tBHP),^9, 10, 11, 12, 13, 14, 15, 16^ with only few studies suggest necrosis as a mechanism for RPE death.^17, 18^ Elucidating the RPE cell death mechanism is imperative to understand AMD pathogenesis and will be instrumental for designing targeted therapy for late-stage AMD, especially GA.

Two major types of cell death, apoptosis and necrosis, have been delineated in response to oxidative stress.^19^ Apoptosis is characterized by cytoplasm shrinkage, chromatin condensation and fragmentation, apoptotic body formation, caspase activation and maintenance of the plasma membrane. Necrosis, used to be considered as passive, unregulated form of cell death, was recently found to be a regulated process mediated by receptor-interacting protein kinases (RIPKs).^20^ In contrast to apoptosis, necrosis is characterized by ATP depletion, increase in cell volume and rupture of the plasma membrane. Moreover, apoptosis is anti-inflammatory, whereas necrosis triggers inflammation and immune response. Necrotic cells can release multiple pro-inflammatory factors, including high mobility group protein B1 (HMGB1) proteins, to activate inflammatory response.^21^ Besides apoptosis and necrosis, autophagy and pyroptosis are also linked to cell death. Autophagy involves bulk degradation of intracellular materials in response to stress and generally has pro-survival roles in disease. Overactive autophagy may be cytotoxic.^22^ Autophagy is characterized by the formation of autophagosome containing lipidated microtubule-associated protein light chain 3 (LC3). Pyroptosis is a pro-inflammatory programmed cell death uniquely dependent on caspase-1.^23^

In an effort to clarify the mechanism of oxidative stress-induced RPE cell death, we found that molecular features of apoptosis, autophagy or pyroptosis were not observed in RPE cells in response to H_2_O_2_ or tBHP treatment. Instead, ATP depletion, RIPK3 aggregation, membrane breakdown and HMGB1 release, which are cardinal features of necrosis, were detected in the treated cells. Moreover, inhibition of RIPK activity by necrostatins or *RIPK3* mRNA level by siRNAs largely rescued oxidative stress-induced RPE death. Our data provide compelling evidence that necrosis is a major type of cell death in response to oxidative stress, highlighting the potential of therapeutic targeting RPE cell necrosis in GA.

## Results

### Evidence against H_2_O_2_ (or tBHP)-induced apoptosis in RPE cells

**Figure 1:**
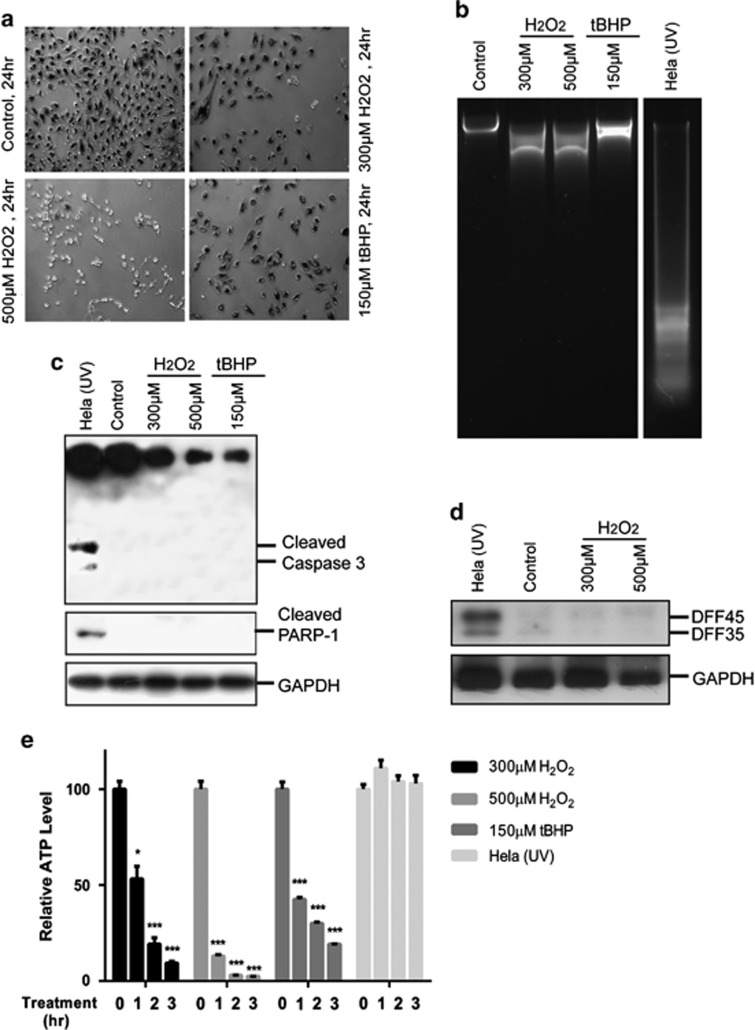
Lack of apoptotic hallmarks in ARPE-19 cells subjected to oxidative stress. (**a**) Light microscopy pictures of MTT crystals in ARPE-19 cells showing decrease in cell number and viability of ARPE-19 cells treated with 300 or 500 *μ*M of H_2_O_2_, or 150 *μ*M of tBHP. Test was performed at 24 h after inducing oxidative stress. (**b**) Analyses of DNA fragmentation in ARPE-19 cells treated with 300 or 500 *μ*M of H_2_O_2_, or 150 *μ*M of tBHP. DNA from UV-irradiated Hela cells was used as a positive control for apoptosis. DNA was purified from both dead and live cells 24 h after inducing cell death and analyzed by gel electrophoresis. Partial DNA fragmentation in ARPE-19 cells is visible as a smear in the agarose gel. (**c**) Western blotting detection of cleaved caspase-3 and PARP products as a result of cell death. 25 *μ*g of total cell extract was prepared from both dead and live cells 24 h after subjecting cells to oxidative stress (ARPE-19) or UV irradiation (Hela). GAPDH served as loading control. (**d**) Western blot measurement of DFF45 level in control ARPE-19 cells and cells subjected to oxidative stress. Of note, the DFF antibody recognizes both DFF45 and DFF35. Hela cells were used as reference. GAPDH served as loading control. (**e**) Analyses of ATP levels in ARPE cells subjected to oxidative stress. ATP level was measured by recording luminescence in indicated time points after treating cells with 300 or 500 *μ*M of H_2_O_2_, or 150 *μ*M of tBHP. UV-irradiated Hela cells were used as a control for apoptosis. *P<0.05; ***P<0.001

We began with validating the system for studying oxidative stress-induced RPE cell death. Sub-confluent ARPE-19 cells were treated with H_2_O_2_ or tBHP, and cell viability was measured by MTT assay 24 h later. RPE cells show increasing rate of cell death upon increasing H_2_O_2_ or tBHP treatment. In line with the published results, low concentrations of H_2_O_2_ (<100 *μ*M) or tBHP (<50 *μ*M) had little effect on RPE viability. However, RPE cell survival dropped to ∼40% and ∼18% when cells were exposed to 300 and 500 *μ*M H_2_O_2_, and ∼30% and ∼10% of RPE cells survived at 150 and 250 *μ*M tBHP treatment, respectively (Figure 1a and Supplementary Figure 1). These validate the system to study oxidative stress-induced RPE cell death.

Morphologically, dying ARPE-19 cells from H_2_O_2_ (or tBHP) showed cell shrinkage followed by rounding up similar to what occurs in oncosis (Supplementary Figure 2). These cells did not exhibit typical apoptotic DNA chromatin condensation by 4,6-diamidino-2-phenylindole (DAPI) staining, although partial chromatin condensation was observed (Supplementary Figure 3). Furthermore, no classic apoptotic DNA laddering was observed, although partial DNA degradation was detected (Figure 1b). These argue against apoptosis as a major type of RPE cell death treated with H_2_O_2_ or tBHP. The execution of apoptosis requires the activation of the effector caspases such as caspase 3, which can be shown by its cleavage and also the cleavage of its downstream substrates including poly(ADP-ribose) polymerase (PARP).^24^ Caspase-3 zymogen expression was decreased in H_2_O_2_ (or tBHP)-treated RPE cells by western blot analyses. However, the cleaved form of caspase-3 and PARP was not detected, which contrasts to that occurs in the UV-induced Hela cells and indicates a lack of caspase-3 activation (Figure 1c). To further examine the mechanism for the lack of DNA fragmentation in RPE cells, western blot analysis was used to measure the protein level of apoptotic endonuclease DNA fragmentation factor (DFF). DFFs form heterodimeric complex that mediates regulated apoptotic chromatin condensation and DNA fragmentation, and DFF40 is released and activated upon DFF45 cleavage by caspase-3.^25^ Compared with Hela cells, DFF protein expression was barely detectable in RPE cells regardless of H_2_O_2_ treatment (Figure 1d). These results suggest intrinsic molecular impairments in apoptotic pathways in RPE cells that prevent oxidative stress-induced apoptosis.

Another determinant of cell death by apoptosis versus necrosis is the levels of intracellular ATP.^26^ Apoptosis contains several ATP-dependent steps, and intracellular ATP levels remain largely unchanged until the very end of the process, whereas necrosis occurs under intracellular ATP depletion. Rapid depletion of ATP in ARPE-19 cells was observed with ∼90% and ∼97% depletion detected by 300 and 500 *μ*M H_2_O_2_ treatment, respectively, in the course of 3 h (Figure 1e). tBHP also caused depletion of the intracellular ATP, although at a slower pace. UV-induced apoptotic control Hela cells showed minimal ATP level change (Figure 1e).

Taken together, cardinal features of apoptosis, including DNA fragmentation, caspase 3 cleavage and maintaining of intracellular ATP level, were not observed in dying RPE cells in response to oxidative stress, indicating that apoptosis is not a major mechanism of oxidative stress-induced RPE death.

### Inhibition of RPE cell death by RIPK inhibitors necrostatins but not caspase inhibitor z-VAD

**Figure 2:**
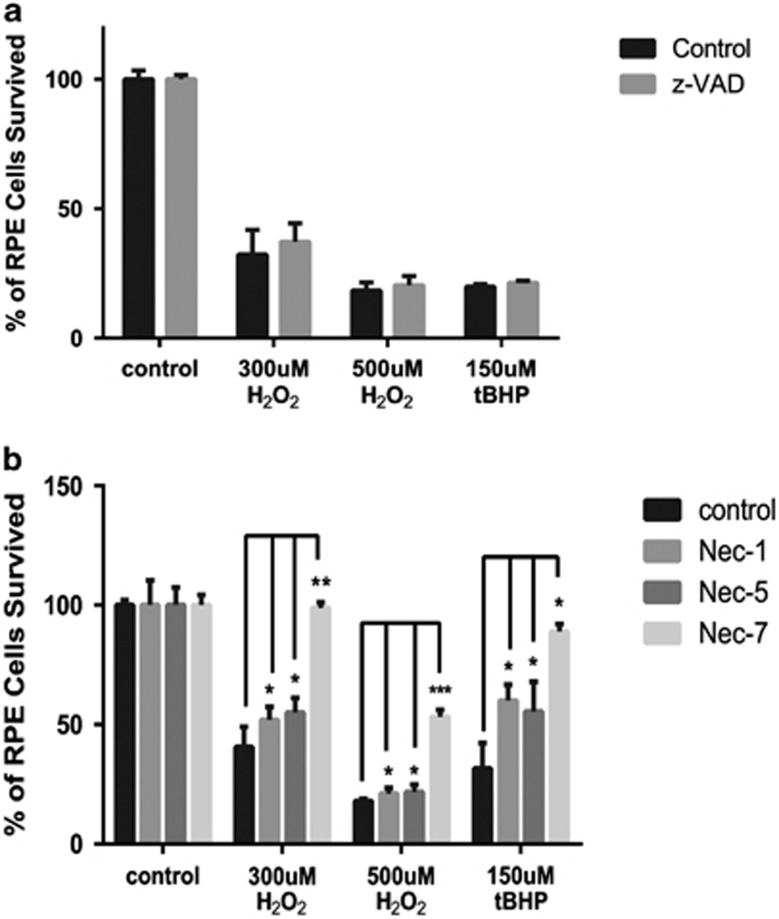
Necrostatins but not z-VAD can rescue ARPE-19 from cell death induced by oxidative stress. (**a**) ARPE-19 cells were treated with 33 *μ*M of z-VAD for 24 h before inducing oxidative stress with different concentrations of H_2_O_2_ or tBHP. Cell viability was measured by MTT assay at 24 h after induction of oxidative stress. (**b**) MTT assay after ARPE-19 cells were treated with 33 *μ*M necrostains 1, 5 or 7 for 24 h before inducing oxidative stress as described above. *P<0.05; **P<0.01; ***P<0.001

Necrosis has been recently recognized as a regulated type of cell death in response to insults.^20^ RIPK3 is essential for necrosis, whereas RIPK1 is critical for both necrosis and apoptosis.^27, 28^ To probe whether necrosis or apoptosis dominates in RPE cells in response to oxidative stress, RPE cells were pre-incubated with z-VAD (pan-Caspase inhibitor, 33 *μ*M) or different necrostatins (Nec, RIPK1 inhibitor, 33 *μ*M) for 24 h prior inducing oxidative stress. Nec-1 is the most common inhibitor of necrosis that targets RIPK1; Nec-5 is necrosis inhibitor that inhibits RIPK1 indirectly;^29^ whereas Nec-7 targets RIPK1-independent necrosis pathways.^30^ Consistent with a lack of apoptosis in RPE cells in response to H_2_O_2_ or tBHP, caspase inhibitor z-VAD did not affect RPE death (Figure 2a). The efficiency of z-VAD was confirmed using UV-treated Hela cells (Supplementary Figure 4). However, a significant increase in survival was observed in RPE cells when pretreated with either of the necrostatins (Figure 2b). Among them, Nec-7 appeared to have better protective effect than Nec-1 or Nec-5. This observation suggests that necrosis is a predominant form of RPE cell death in response to oxidative stress.

### Membrane changes in H_2_O_2_ (or tBHP)-treated RPE cells

**Figure 3:**
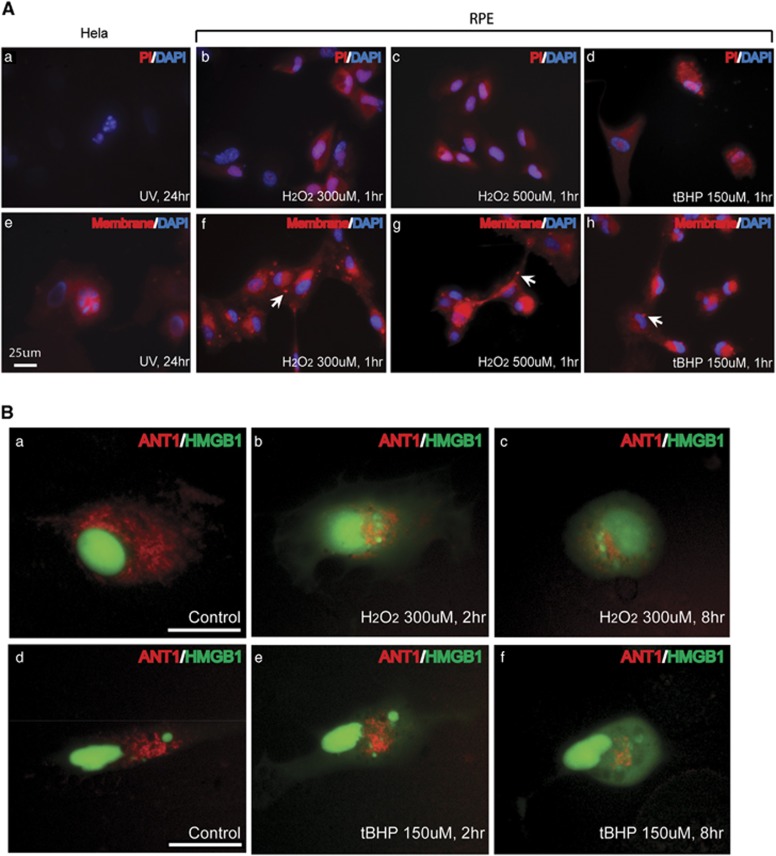
Membrane changes during RPE cell death by oxidative stress. (**A**) Cell membrane changes in non-fixed ARPE-19 cells in the indicated conditions shown by staining with PI (a–d) and CellMask Orange Plasma Stain (e–h). Cell nucleus was stained with DAPI. Arrows marked the membrane blebs formed in the treated cells. Scale bar equals 25 *μ*m. (**B**) Mitochondrial network and nuclear envelope permeability were tracked by YFP (green) and RFP (red) signal after ARPE-19 cells were transfected with HMGB1-YFP and ANT1-RFP expression plasmids and treated H_2_O_2_, or tBHP for indicated time. a and d are untreated control cells. b and c, e and f are cells treated with H_2_O_2_ and tBHP respectively. Scale bar equals 25 *μ*m

In contrast to apoptosis, necrotic cells show cellular membrane permeability as indicated by propidium iodine (PI) staining. Unfixed RPE cells were incubated with PI after H_2_O_2_ or tBHP treatment to examine membrane permeability. Cellular PI staining was not observed in apoptotic Hela or untreated RPE cells (Figure 3A (a) and data not shown). Cytoplasmic PI staining was observed in RPE cells as early as 1 h after H_2_O_2_ or tBHP treatment (Figure 3A (b–d)). To gain more insight into the cell membrane change during RPE cell death, the cellular membrane was stained with CellMask Orange Stain. Membrane blebs were observed in RPE cells as early as 1 h after treatment, but not in apoptotic Hela cells (Figure 3A (e–h)). These membrane blebs did not contain DAPI-stained chromatin indicating they are not apoptotic bodies. High mobility group proteins are essential part of chromatin structure. During necrosis, HMGB1 protein is passively released from nucleus and secreted to extracellular matrix.^31^ During apoptosis, HMGB1 is tightly bound to DNA and sequestrated inside apoptotic bodies.^32^ When plasmid expressing HMGB1-YFP was transfected into RPE cells, it was located inside the nucleus (Figure 3B (a, d)). Within 2–8 h of H_2_O_2_ or tBHP treatment, HMBG1 was released to the cytosol (Figure 3B (b, c and e, f)). Under higher concentration of H_2_O_2_ (500 *μ*M), HMGB1 was released to the cytosol within first 2 h.

Mitochondrial fragmentation is involved in both apoptosis and necrosis.^33, 34^ Adenine nucleotide translocase (ANT) 1 is an important structural component of the mitochondrial permeability transition pore.^35^ Mitochondria morphology was visualized by transfecting RPE cells with plasmid expressing RFP-ANT1. Normally, mitochondria form tubular network in the cytosol as indicated by RFP staining (Figure 3B (a, d)). At 2–8 h after H_2_O_2_ (300 *μ*M) or tBHP (150 *μ*M) treatment, fragmentation and degeneration of mitochondrial network became obvious, correlating with nuclear membrane leakage shown by HMBG1 release (Figure 3B (b, c and e, f)). Under high concentration of H_2_O_2_, the disruption of mitochondrial network and release of HMGB1 occurred within first 2 h.

Intracellular PI staining, membrane blebbing and rupture and HMGB1 nuclear release in H_2_O_2_ (or tBHP)-treated RPE cells indicate that integrity of nuclear and cytoplasmic membrane is compromised by oxidative stress, further supporting that necrosis, rather than apoptosis, is a major mechanism of RPE cell death in response to oxidative stress.

### RIPK3 aggregation and its requirement for RPE cell necrosis in response to oxidative stress

**Figure 4:**
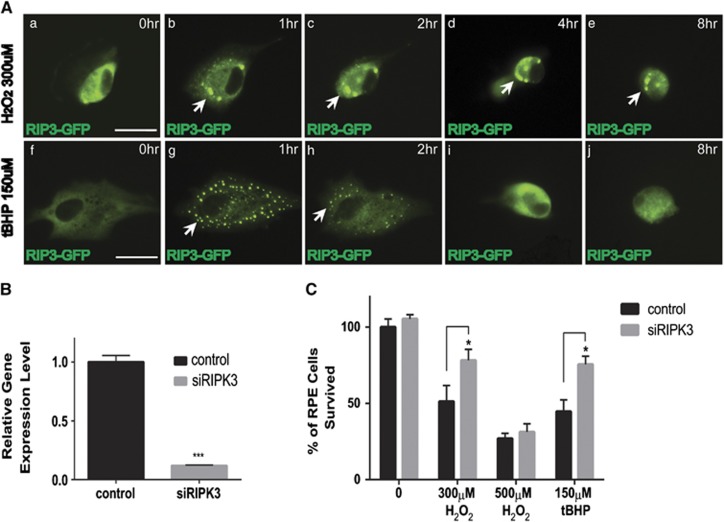
RIPK3 activation and its requirement in ARPE-19 cell death in response to oxidative stress. (**A**) Sequential images showing the distribution and aggregation of RIPK3 in ARPE-19 cells transfected with RIPK3-GFP expression plasmid and treated with 300 *μ*M H_2_O_2_ (a–e) or 150 *μ*M tBHP (f–j) for indicated times. Scale bar equals 25 *μ*m. The arrows denote the RIPK3 aggregates in the treated cells. (**B**) Knockdown of RIPK3 by transfection with RIPK3 siRNAs shown by real-time qPCR. ***P<0.001. (**C**) Rescue of RPE cell death in RIPK3 siRNA-transfected cells at 24 h after H_2_O_2_ (300 *μ*M) or tBHP (150 *μ*M) treatment. *P<0.05. Results from 500 *μ*M H_2_O_2_ are not statistically significant

RIPK3 is normally uniformly distributed in the living cells, and forms discrete punctate as necrosis processes.^36^ To further confirm whether necrosis is a major mechanism for oxidative stress-induced RPE death, RIPK3-GFP expression plasmid was transfected into RPE cells, and its cellular distribution was visualized by GFP signal. Compared with the evenly cytosolic distribution in the controls, RIPK3 punctuates formed in the cell periphery upon H_2_O_2_ or tBHP treatment (Figure 4A (a, b and f, g)). This occurred within the first hour and reached its peak at about 1 h and then slowly diminished (Figure 4A (c–e and h–j)). Compared with H_2_O_2_-treated cells, RIPK3 aggregates in tBHP-treated cells were smaller and more distinct. We next examined whether RIPK3 is required for RPE necrosis in response to oxidative stress. To do so, two sets of siRNA against *RIPK3* were transfected into ARPE-19 cells. By real-time RT-PCR (Figure 4B), maximal knockdown efficiency (more than 90%) was achieved when two sets of *RIPK3* siRNAs were combined in the transfection. *RIPK3* knockdown dramatically prevented RPE cell death in response to H_2_O_2_ (300 *μ*M) or tBHP (150 *μ*M), although its effect was not significant with more harsh H_2_O_2_ (500 *μ*M; Figure 4C). Compared with the 40–50% survival in the treated control cells, RPE survival increased to about 80% upon RIPK3 knockdown. These data further confirm the necrotic nature of RPE death and indicate a critical role for RIPK3 in oxidative stress-induced RPE necrosis.

### Inflammatory gene expression induced by H_2_O_2_ (or tBHP)-induced RPE cell death

**Figure 5:**
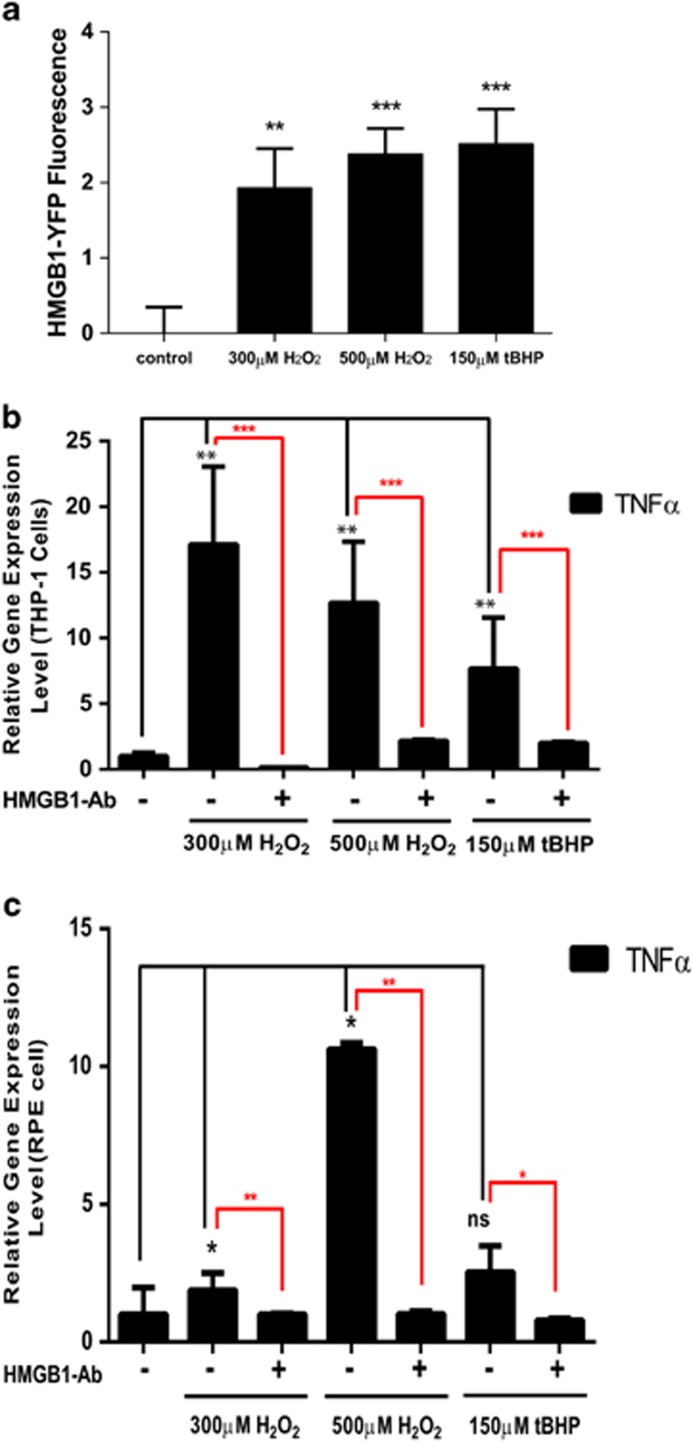
Dying ARPE-19 cells from oxidative stress induce the expression of pro-inflammatory genes in healthy cells. (**a**) HMHB1 released to the cell medium as measured by YFP fluorescence in ARPE-19 cells transfected with HMGB1-YFP expression plasmid and treated with 300 or 500 *μ*M H_2_O_2_, or 150 *μ*M tBHP for 24 h. (**b**) Inflammatory gene *TNFα* expression measured by real-time RT-PCR in differentiated THP-1 cells after 24 h treatment with conditioned medium collected from dying ARPE-19 cells subjected to oxidative stress. Gene expression in THP-1 cells treated with cell medium from healthy ARPE-19 cells was used as control. **P<0.01; ***P<0.001. (**c**) Inflammatory gene *TNFα* expression in healthy ARPE-19 cells after 24 h treatment with conditioned medium collected from dying ARPE-19 cells subjected to oxidative stress. Gene expression in ARPE-19 cells treated with cell medium from healthy ARPE-19 cells was used as control. *P<0.05; **P<0.01; NS, nonsignificant

HMGB1 protein released from necrotic cells has been shown to induce inflammatory response.^31^ Consistent with the observed HMGB1 release, we found significant HMGB1-YFP signal in the medium of HMGB1-YFP-transfected RPE cells treated with H_2_O_2_ or tBHP, which was not observed in the medium from the control cells (Figure 5a). Next, we tested the ability of the cell medium to induce inflammatory gene expression in healthy activated macrophages (THP-1 cells) and healthy RPE cells. Conditioned cell medium was collected at 24 h after H_2_O_2_ or tBHP treatment, and used to treat normal THP-1 or RPE cells. *TNFα* expression was induced significantly by medium from either H_2_O_2_- or tBHP-treated RPE cells when normalized to the control medium, with ∼17-fold by 300 *μ*M H_2_O_2_ and ∼7-fold by 150 *μ*M tBHP (Figure 5b). Significant upregulation of *TNFα* was also observed in healthy RPE cells by the medium from the dying cells treated with H_2_O_2_, although the results from tBHP were not statistically significant (Figure 5c). More importantly, when HMGB1-depleted medium was used, the induction of *TNFα* by the medium was almost blunted (Figures 5b and c). These data support that HMGB1 released from necrotic RPE cells has a critical role in inducing inflammatory gene expression, further corroborating the necrotic nature of RPE cell death.

### Lack of pyroptosis or autophagy in RPE cells treated with H_2_O_2_ or tBHP

**Figure 6:**
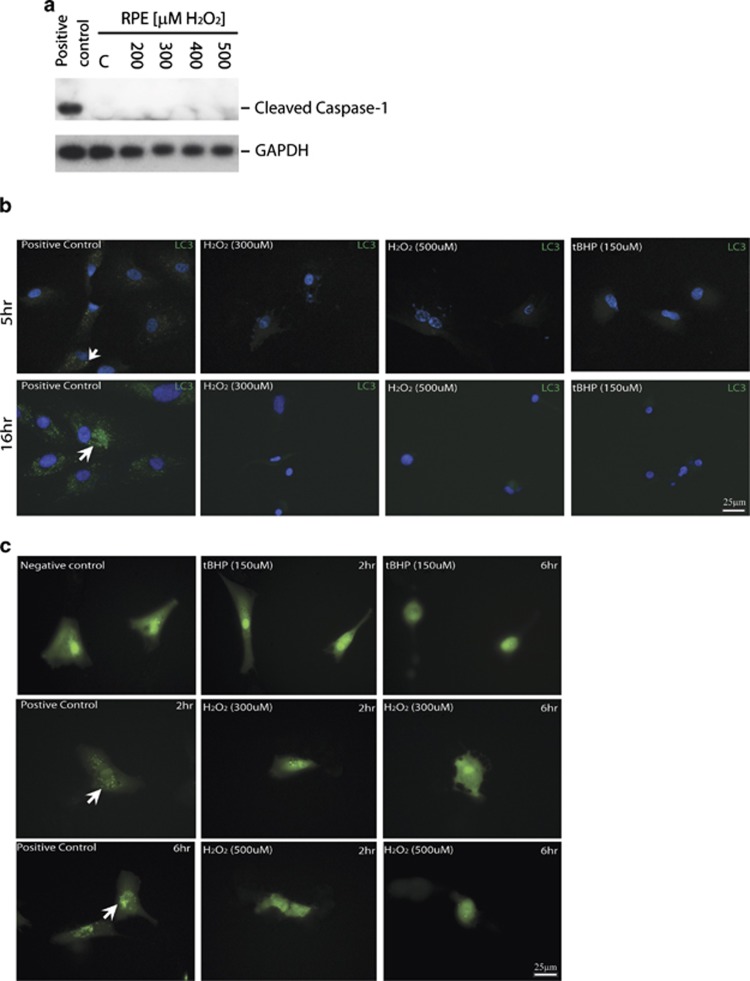
No evidence of pyroptosis or autophagy in ARPE-19 cells in response to oxidative stress. (**a**) No evidence of caspase-1 activation as measured by western blot analysis in ARPE-19 cells at 24 h after treatment with 300 or 500 *μ*M of H_2_O_2_. Caspase 1 activation in ARPE-19 transfected with Alu RNA was used as positive control. (**b**) Lack of autophagosomes by LC3B antibody in ARPE-19 subjected to oxidative stress for indicated times. Choloquine (100 *μ*M)-treated cells were used as positive controls. Scale bar equals 25 *μ*m. Arrows point to the LC3 aggregates in the positive controls. (**c**) Lack of autophagosomes in LC3-GFP-transfected ARPE-19 cells treated by 300 or 500 *μ*M of H_2_O_2_, or 150 *μ*M of tBHP. LC3 distribution in the cells was visualized by GFP fluorescent signal. Choloquine (100 *μ*M)-treated cells were used as positive controls. Scale bar equals 25 *μ*m. Arrows point to the LC3 aggregates in the positive controls

Pyroptosis and autophagic cell death are alternative types of cell death in response to stress. To determine whether pyroptosis and autophagy occur during H_2_O_2_ or tBHP-induced RPE death, caspase-1 activation was examined by western blot analyses, whereas autophagy was monitored by LC3 staining and a LC3-GFP indicator. Caspase-1 activation was not observed in RPE cells treated with different concentrations of H_2_O_2_ (Figure 6a). As control, caspase-1activation was detected in RPE cells transfected with Alu RNA^37^ (Figure 6a). By LC3 antibody staining, in contrast to the positive control, LC3 punctate was not observed in RPEs at 5 or 16 h after H_2_O_2_ or tBHP treatment (Figure 6b). Consistently, the autophagic dynamic LC3 turnover indicated by LC3-GFP signal was not observed in LC3-GFP-transfected RPEs after H_2_O_2_ or tBHP treatment (Figure 6c). These data suggest that pyroptosis or autophagic cell death is not a major mechanism for oxidative stress-induced RPE death.

### Evidence of necrosis in RPE cells under prolonged low oxidative stress

**Figure 7:**
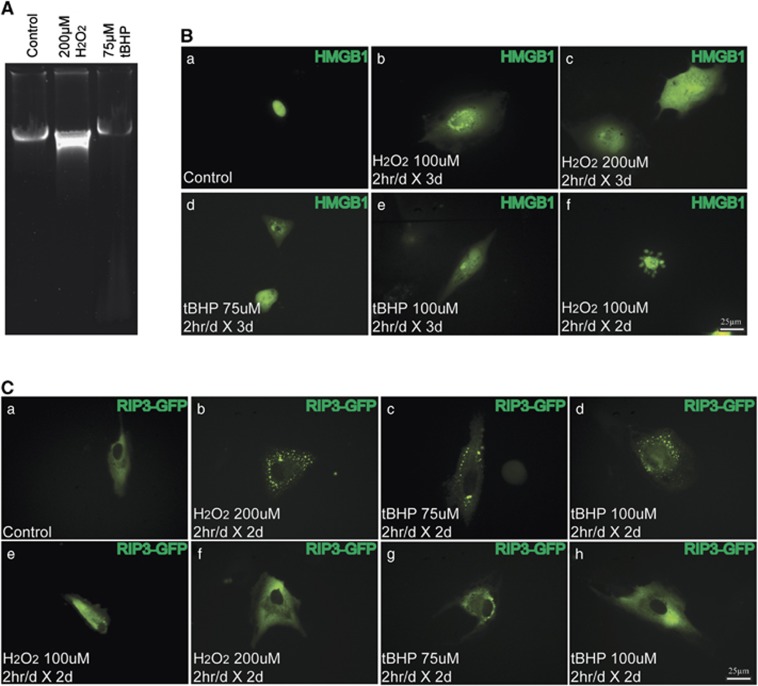
Detection of necrosis in ARPE-19 cells in response to prolonged low oxidative stress. (**A**) Lack of apoptotic DNA degradation in APRE-19 cells subjected to low oxidative stress (2 h/day for 4 days). (**B**) Increase in nuclear membrane permeability in HMGB1-YFP-transfected ARPE-19 cells subjected to prolonged low oxidative stress. HMGB1-YFP was localized in nucleus in non-treated cells (a), but released to the cytoplasm under the indicated treatments (b–e). A small percentage of cells seem to undergo apoptosis as shown by the apoptotic bodies in f. (**C**) RIPK3 aggregation detected in RIPK3-GFP-transfected ARPE-19 cells upon prolonged low oxidative stress. No obvious RIPK3 aggregation was observed in control cells or cells treated with 100 *μ*M H_2_O_2_ for 2 days (a and e). Pattern of RIPK3 aggregation (b–d) and normal RIPK3 distribution (f–h) were observed in cells treated with 200 *μ*M H_2_O_2_, 75 *μ*M tBHP or 100 *μ*M tBHP for 2 days

To further examine the mechanism of RPE cell death under prolonged low oxidative stress, ARPE-19 cells were treated with 100 or 200 *μ*M H_2_O_2_, or 75 or 100 *μ*M tBHP 2 h/day for up to 4 days.^38^ ARPE-19 cells showed ∼80% survival after 4 days of short-term 100 *μ*M H_2_O_2_ or 75 *μ*M tBHP treatment, which decreased to ∼40% and ∼70% when the concentration increased to 200 and 100 *μ*M, respectively (Supplementary Figure 5). Consistently, no apoptotic DNA laddering was observed in the treated cells, supporting that apoptosis is not a major mechanism for RPE cell death under these conditions. To further test whether necrosis occurs in these cells, similar HMGB1-YFP transfection was performed. Compared with nuclear localization of HMGB1 in the controls, HMGB1 release into the cytoplasm was readily detected at 2–3 days after low-concentration H_2_O_2_ or tBHP treatment (Figure 7B (a–e)). Of note, few cells appeared to undergo apoptosis as shown by the HMGB1-positive apoptotic bodies (Figure 7B (f)). By using a RIPK3-GFP indicator, no obvious RIPK3 aggregation was observed in control cells or cells treated with 100 *μ*M H_2_O_2_ for 2 days (Figure 7C (a, e)). In line with the less severe cell death under these conditions, both RIPK3 aggregation (Figure 7C (b–d)) and normal RIPK3 distribution (Figure 7C (e–h)) were observed in cells treated with 200 *μ*M H_2_O_2_, 75 *μ*M tBHP or 100 *μ*M tBHP for 2 days. Taken together, our results indicate that necrosis also accounts for the majority of RPE cell death under prolonged low oxidative stress.

## Discussion

The current paradigm is that RPE cells die mainly from apoptosis in AMD.^8^ Here we provide the following observations of RPE cell death in response to oxidative stress: (1) lack of chromatin condensation and DNA fragmentation; (2) absence of activated caspase 3 and the cleavage of its substrate PARP; (3) rapid depletion of intracellular ATP; (4) rescue of RPE death by RIPK inhibitor necrostains but not caspase inhibitor z-VAD; (5) nuclear and cytoplasm membrane permeability change and breakdown shown by PI staining and HMGB1 release; (6) RIPK3 aggregation and rescue of RPE cell death by RIPK3 knockdown; (7) TNF*α* induction by cell medium from dying RPE cells subject to oxidative stress; (8) lack of pyroptosis and autophagy. Taken together, our data argue against apoptosis as a major mechanism of RPE cell death, and unequivocally establish necrosis as a major mechanism of RPE cell death in response to oxidative stress.

### Apoptosis is not a main mechanism for oxidative stress-induced RPE cell death

Historically, TUNEL assay has been used to probe apoptosis based on its detection of nicked DNA, which attributes to the current paradigm that RPE and photoreceptor die from apoptosis in AMD. However, this assay fails to discriminate apoptotic from necrotic cells given that both have free DNA ends. Although photoreceptors are known to die from apoptosis in AMD, the mechanism of RPE cell death in AMD is becoming controversial. We performed systematic analysis of ARPE-19 cell death induced by oxidative stress using two different concentrations of H_2_O_2_ (300 and 500 *μ*M) and tBHP (150 *μ*M). ARPE-19 clearly lacked typical apoptotic DNA laddering when treated with either oxidative stress, or even prolonged low oxidative stress. We indeed observed some degree of random chromatin degradation, more in H_2_O_2_ than in tBHP-treated cells. We did not observe cleavage of the apoptosis effector enzyme caspase 3 in RPE cells under either stress, consistent with a lack of its downstream apoptosis-specific PARP cleavage. Induction of apoptosis triggers casapse-3-mediated DFF45 cleavage and release of DFF40 subunit leading to DNA fragmentation. Consistent with the lack of apoptotic DNA laddering, we found that DFF45/40 expression is much lower than in Hela cells. These results indicate intrinsic impairments of apoptotic pathway in RPE cells, consistent with a recent report that RPE cells have low caspase 8 expression and fail to undergo TNF*α*-induced apoptosis.^39^

### Morphological and molecular feature of RPE necrosis in response to oxidative stress

Typical necrosis is characterized by ATP depletion, increased cell volume and rupture of the plasma membrane. We tested whether caspase inhibitor or RIPK inhibitor can rescue RPE death from H_2_O_2_ or tBHP, and found that caspase inhibitor z-VAD failed to rescue oxidative stress-induced RPE death but RIPK inhibitor Nec-7 could, suggesting that RPE cells die mainly from necrosis. HMGB1 binds to DNA tightly during apoptosis, but is passively released from nucleus and secreted during necrosis. We observed HMGB1 nuclear release and secretion in the treated RPE cells, consistent with an increase in membrane permeability and cell rupture in those cells. Furthermore, rapid intracellular ATP depletion was also detected in the treated RPE cells, which does not normally occur in apoptosis. These results further confirm the necrotic feature of RPE death in response to oxidative stress, but also suggest that it might be atypical necrosis as cell swollen was not observed.

Molecularly, RIPK3 activation shown by RIPK3 aggregation is a hallmark of necrosis.^28, 36, 40^ Caspase 8 is a switch between apoptosis and necrosis and cleaves RIPK3, therefore favoring apoptosis over necrosis.^41^ As we discussed, low level of caspase 8 in RPE cells may tip the balance in RPE cells from apoptosis to necrosis.^39^ By using a RIPK3-GFP indicator, we found distinct RIPK3 aggregation in oxidative stress-treated RPE cells, which demonstrates that RIP3 kinase is activated when necrosis is triggered as a result of oxidative stress in RPE cells. More importantly, RIPK3 knockdown significantly rescued RPE cell death upon oxidative stress, indicating that RIPK3 is critical for oxidative stress-induced RPE necrosis. Moreover, key features of pyroptosis and autophagy were not observed during oxidative stress-induced RPE death. These results further strengthen our hypothesis that necrosis is a major type of oxidative stress-induced RPE cell death, consistent with a very recent report that double-strand RNA induces RPE necrosis, but not apoptosis *in vivo*.^42^

### Inflammatory nature of RPE necrosis by oxidative stress

In contrast to apoptosis, necrosis is generally believed to trigger inflammation and immune response. We found that HMGB1 is released from oxidative stress-treated RPE cells. HMGB1 protein released from necrotic cells is known to induce inflammatory response.^31^ Consistently, we found inflammatory gene *TNFα* is induced in macrophages and also healthy RPE cells by cell medium from the dying cells subjected to oxidative stress. These results suggest that necrotic RPE cells can induce chronic inflammatory response in the neighboring cells. As AMD pathogenesis has strong inflammatory and immune components, our findings that RPE cells die from necrosis and induce inflammatory gene expression may provide a mechanism that triggers inflammation and immune response in AMD, consistent with the critical role of RPE cells in AMD etiology.

### Therapeutic implications and future directions

Currently, the mechanism for AMD pathogenesis remains unclear, and there is no cure for dry AMD, especially GA. Extensive loss of RPE cells (RPE atrophy) was observed in late dry AMD, which accounts for photoreceptor apoptosis and vision loss in AMD. Our findings that RPE cells die mainly from necrosis in response to oxidative stress and induce inflammatory gene expression in the healthy RPE cells provide a novel mechanism for AMD pathogenesis. Our results suggest that targeting necrosis and/or oxidative stress may be a viable approach for dry AMD therapeutics. More specifically, Nec-7, or RIPK3 inhibitors, or antibodies to HMGB1 may have therapeutic value in GA. Future work should focus on re-evaluation of RPE cell death mechanism in AMD animal models and human AMD patients, as well as testing targeted anti-necrotic therapy for the devastating blinding AMD disease.

## Materials and Methods

### Cell culture and treatments

Human Retina Pigment Epithelium cell line (ARPE-19, CLR-2302, ATCC, Manassas, VA, USA) was cultured in DME/F-12 medium (HyClone, Logan, UT, USA) supplemented with 10% FBS (HyClone) and 1 × penicillin-streptomycin solution (HyClone) at 37 °C in 5% CO_2_. Solutions of H_2_O_2_ (Sigma-Aldrich) and *tetr*-Butyl hydroperoxide (Sigma-Aldrich, St. Louis, MO, USA) were freshly prepared in growth culture medium before adding to the cell culture. For prolonged low oxidative stress, RPE cells were treated for 2 h/day for up to 4 days with tBHP or H_2_O_2_ solutions. Hela cells were UV irradiated (254 nm light at a 60 J/m^2^ dose) and collected at up to 24 h later to detect apoptosis. Human monocytic cell line THP-1 (ATCC) cells were cultured and differentiated into macrophage as described.^43^ To study the effect of cell medium from dying RPE cells on healthy RPE or THP-1 cells, ARPE-19 were treated with 300, 500 *μ*M of H_2_O_2_ or 150 *μ*M tBHP for 24 h. Conditioned medium collected was then added to healthy ARPE-19 or activated THP-1 cells to substitute their normal growth medium. The effect of conditioned medium on inflammatory gene expression in healthy cells was analyzed at 24 h later by real-time qPCR. For RPE cell treatment with apoptosis inhibitor z-VAD (Sigma-Aldrich), or necrosis inhibitors necrostatin-1, -5, -7 (Enzo Life Sciences, Farmingdale, NY, USA), cells were treated with 33 *μ*M of each inhibitor for 24 h before subjecting to oxidative stress.

### MTT and ATP assay

To study cell viability, about 5000 ARPE-19 cells were cultured overnight in 96-well plates. After treatment with H_2_O_2_ or tBHP for 24 h, the cells were incubated with 1 mg/ml of MTT reagent (Sigma-Aldrich) for 3 h under standard cell culture conditions. Once MTT crystals were developed, pictures were taken under a light microscope. MTT crystals were then dissolved in DMSO (Sigma-Aldrich) and quantified by measuring absorbance at 540 nm. To measure cellular ATP level, CellTiter-Glo (Promega, Madison, WI, USA) was used according to manufacturer's directions. In brief, after equilibrating to room temperature, equal volume of the CellTiter-Glo reagent was added to the cell medium of H_2_O_2_ or tBHP-treated cells. The fluorescence signal was read using a Perkin Elmer Victor X3 Multilabel Plate Reader (Perkin Elmer, Waltham, MA, USA).

### Cell transfection

Cell transfection was performed using Lipofectamine LTX unless otherwise indicated (Life Technologies, Carlsbad, CA, USA). Briefly, 1 *μ*g of HMGB1-YFP, ANT1-RFP or RIPK3-GFP plasmid DNA was mixed with 5 *μ*l of Lipofectamine LTX. The complex was added to the ARPE-19 cell cultured in four-chamber glass slide 20 min later. Expression of the recombinant proteins was visualized after 24 h using a fluorescent microscope. For RIPK3 siRNA transfection, two sets of siRNAs targeting RIPK3 were transfected into ARPE-19 cells at 50 nM each using Lipofectamine RNAi MAX (Life Technologies) to ensure efficient RIPK3 knockdown. Sequences for RIPK3 siRNAs are as follows: set 1: sense: 5′-GCGAUAUCCAGGGAGGUCA-3′, antisense 5′-UGACCUCCCUGGAUAUCGC-3′ set 2: sense: 5′-GACAACAACUACUUGACUA-3′, antisense: 5′-UAGUCAAGUAGUUGUUGUC-3′.

### Membrane permeability assay and membrane staining

To analyze membrane permeability, non-fixed ARPE-19 cells subjected to oxidative stress (H_2_O_2_ or tBHP) were stained with the 0.5 *μ*g/ml PI for 5 min. The PI staining solution was prepared in growth medium and added to the cells at 1–12 h after inducing oxidative stress. After removing PI staining solution, cells were washed with PBS and overlaid with mounting medium containing DAPI. To visualize cell membrane, non-fixed ARPE-19 cells were subjected to oxidative stress for 1–12 h and incubated with 0.25 *μ*g/ml CellMask Orange Plasma Stain (Life Technologies) prepared in growth medium for 5 min. The staining solution was then removed and the cells were washed with PBS and overlaid with mounting medium containing DAPI. Cells were immediately analyzed under a fluorescent microscope.

### Protein analyses

Protein levels were analyzed by western blot analysis as described.^44^ Briefly, ARPE-19 cells were treated with 300 or 500 *μ*M H_2_O_2,_ or 150 *μ*M tBHP for 24 h. ARPE-19 cells transfected with Alu RNA was used as positive control for caspase-1 activation.^37^ Alu was cloned by PCR and *in vitro* transcribed, and transfected into cells using a standard procedure. UV-irradiated Hela cells were used a control for apoptosis. Detached cells were collected by centrifugation; cells attached to the plate surface were trypsinized and collected by centrifugation. Both types of cells were mixed together, washed briefly with PBS and resuspended in lysis buffer (50 mM Tris-HCl (pH 7.4), 150 mM NaCl, 1 mM EDTA, 1% Triton X-100) supplemented with protease and phosphatase inhibitors. Protein signals were visualized using SuperSignal West Pico Chemiluminescent Substrate (Thermo Scientific, Waltham, MA, USA). Antibodies used include: rabbit polyclonal anti-caspase 1 (1 : 1000, Millipore, Billerica, MA, USA), rabbit polyclonal anti-caspase 3 (1 : 1000, Cell Signaling, Danvers, MA, USA), mouse monoclonal anti-cleaved PARP (Asp214; 1 : 1000, Cell Signaling), rabbit polyclonal anti-DFF45/DFF35 (1 : 1000, Cell Signaling), mouse monoclonal anti-GAPDH (1 : 2000, Millipore). For the detection of HMHB1-YFP protein in the cell medium, YFP signal was read using a Perkin Elmer Victor X3 Multilabel Plate Reader at 485/535 nm wavelength.

### DNA purification and detection of DNA laddering

To analyze DNA degradation pattern in RPE cells in response to oxidative stress, ARPE-19 cells were treated with 300 or 500 *μ*M H_2_O_2_, or 150 *μ*M tBHP for 24 h. Detached cells were pooled with trypsinization, washed with PBS and resuspended in a lysis buffer (1% SDS, 10 mM EDTA, 1 *μ*g/*μ*l Proteinase K). After incubation for 3 h at 37 °C, DNA was purified by phenol/chloroform extraction. DNA degradation was analyzed by electrophoresis in 1% agarose gel.

### RNA analyses and HMGB1 depletion

Real-time qPCR was used to test the effect of cell medium from dying RPE cells caused by oxidative stress damage on healthy RPE or THP-1 cells. ARPE-19 cells were treated with 300 and 500 *μ*M of H_2_O_2_ or 150 *μ*M tBHP. The conditioned medium was then collected and centrifuged to remove cell debris at 24 h later. To deplete HMGB1 in the medium, anti- HMGB1antibody (Cell Signaling) was added to the purified medium and incubated overnight at 4 °C to deplete HMGB1. Protein A/G Plus-Agarose (Santa Cruz Biotechnology, Santa Cruz, CA, USA) was then added and incubated for 1 h at 4 °C. After removing the agarose beads by centrifugation, the RPE cell medium or HMGB1-depleted RPE cell medium were collected and applied to healthy ARPE-19 cells or THP-1 cells by substituting normal growth medium. The treated ARPE-19 cells or THP-1 cells were collected at 24 h later and the expression of inflammatory gene *TNFα* was analyzed by real-time qPCR. Primers for real-time PCRs include, *CyclophilinA* 5′-CCCGTGTTCTTCGACAT-3′ and 5′-CCAGTGCTCAGAGCACGAAA-3′ *TNFα* 5′-AACCTCCTCTCTGCCATCAA-3′ and 5′-GGAAGACCCCTCCCAGATAG-3′.

### Detection of autophagy

Autophagy was visualized by LC3 staining using a LC3B Antibody Kit (Life Technologies) or transfection of RPE cells with LC3-GFP plasmid. For LC3-GFP transfection, FuGene6 (Roche, Indianapolis, IN, USA) was used. 1 *μ*g of plasmid DNA was mixed with 3 *μ*l of FuGene 6 and added to the cells at 20 min later. The expression and localization of the recombinant protein were visualized at 24 h after transfection plus 0–16 h of H_2_O_2_ or tBHP treatment under a fluorescent microscope. Immunostaning was performed following a standard procedure. Antibodies used include: anti-LC3B antibody (0.5 *μ*g/ml, Life Technologies) and Alexa Fluor 488 anti-rabbit secondary antibody (Life Technologies).

## Abbreviations

AMD: age-related macular degeneration
ANT: adenine nucleotide translocase
DAPI: 4,6-diamidino-2-phenylindole
DFF: DNA fragmentation factor
GA: geographic atrophy
HMGB1: high mobility group proteins B1
H_2_O_2_: hydrogen peroxide
LC3: microtubule-associated protein light chain 3
PARP: poly(ADP-ribose) polymerase
PI: propidium iodine
RIPK: receptor interacting protein kinases
RPE: retinal pigment epithelium
tBHP: *tert*-butyl hydroperoxide
