# Supplementary material for: Induction of necrotic cell death by oxidative stress in retinal pigment epithelial cells
Source: Cell Death Dis. 2013 Dec 12;4(12):e965–. doi: 10.1038/cddis.2013.478 (PMC3877549; doi:10.1038/cddis.2013.478)

Supplemental Data

Supplemental Figure 1

A) ARPE-19 cell viability cells determined by representative by light microscopy (upper panel) MTT crystal (lower panel) pictures after treatment with different concentration of H_2O_2 for 24 hours.

B) ARPE-19 cell viability cells determined by representative by light microscopy (upper panel) MTT crystal (lower panel) pictures after treatment with different concentration of tBHP for 24 hours.

Supplemental Figure 2

Light microscopy pictures showing morphological changes of ARPE-19 cells at 0, 4 and 8 hours after 300□M H_2O_2 (A-C), 500□M H_2O_2 (D-E) or 150□M tBHP (G-I) treatment.

Supplemental Figure 3

DAPI stainig of ARPE-19 cell nuclei at 8 hours after 300□M or 500□M H_2O_2, or 150□M tBHP treatment (C-E). UV-irradiated Hela cells were used as positive control for apoptosis (B). Arrow marked apoptotic chromatin condensation and nuclear fragmentation in (B).

Supplemental Figure 4

Rescue of Hela cell death in response to UV irradiation by z-VAD.

Supplemental Figure 5

ARPE-19 cell survival in response to prolonged low oxidative stress. RPE cells were treated with 100-200µM H_2O_2 or 75-100µM tBHP 2 hours/day for up to 4 days. Cell survival was measured by MTT assay.

Supplemental Figure 1

A

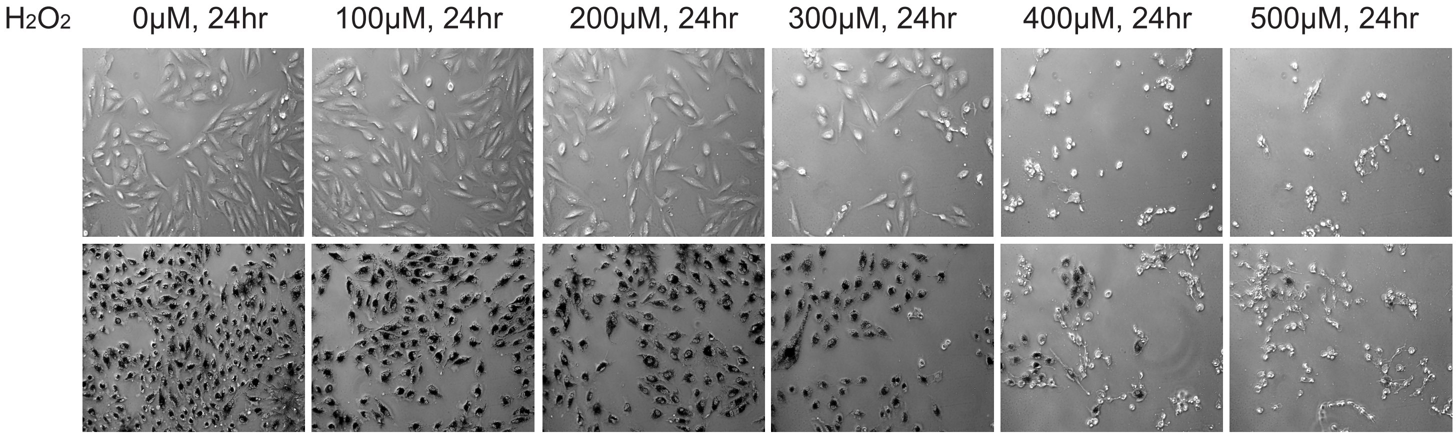

B

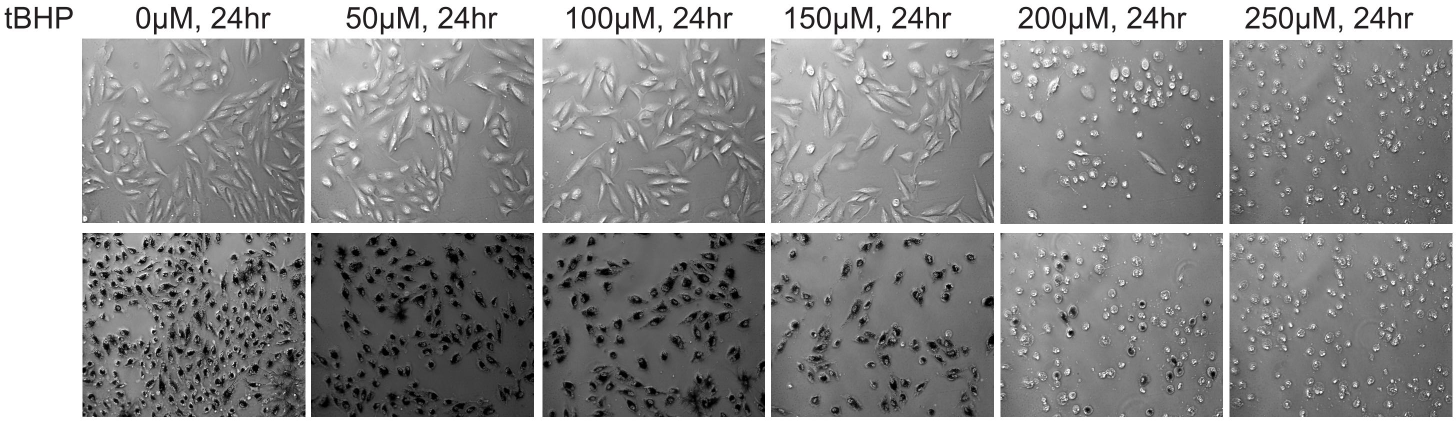

Supplemental Figure 2

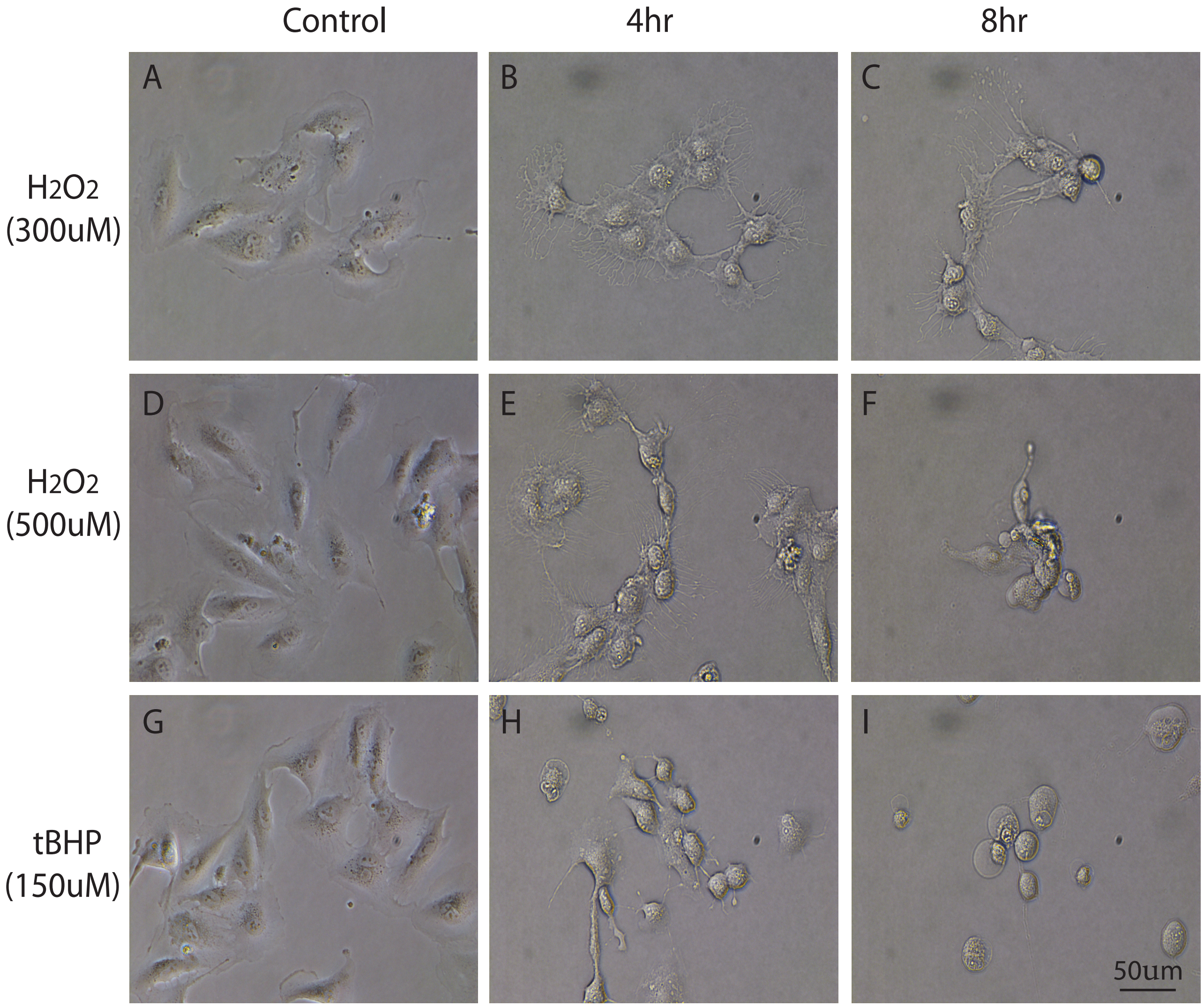

Supplemental Fig. 3

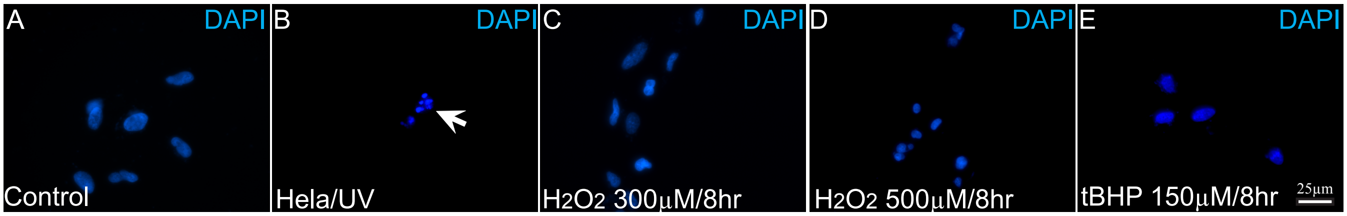

Supplemental Figure 4

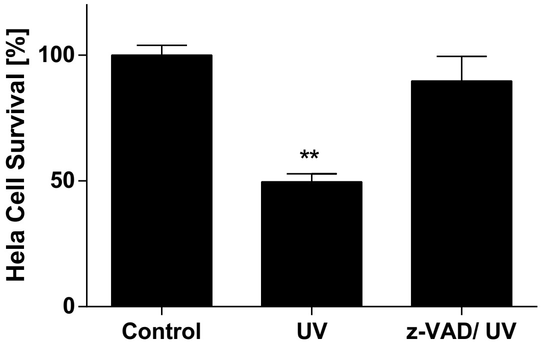

Supplemental Figure 5

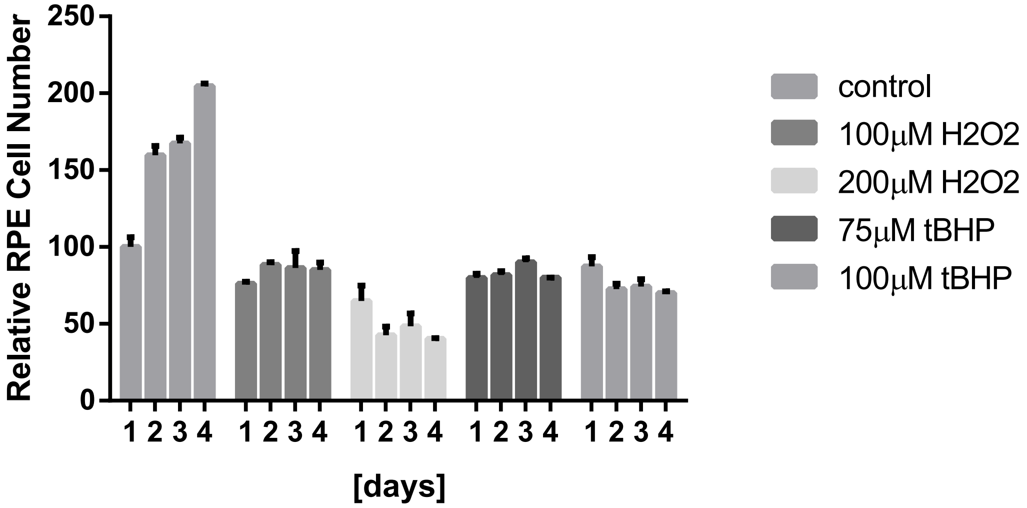

Supplement: Supplementary Data [file cddis2013478x1.pdf]
